# Complement factor I deficiency: a not so rare immune defect. Characterization of new mutations and the first large gene deletion

**DOI:** 10.1186/1750-1172-7-42

**Published:** 2012-06-18

**Authors:** María Alba-Domínguez, Alberto López-Lera, Sofía Garrido, Pilar Nozal, Ignacio González-Granado, Josefa Melero, Pere Soler-Palacín, Carmen Cámara, Margarita López-Trascasa

**Affiliations:** 1 Unidad de Inmunología Hospital Universitario La Paz and Hospital La Paz Health Research Institute (IdiPAZ), Madrid, Spain; 2 Departamento de Pediatría Hospital Universitario 12 de Octubre, Madrid, Spain; 3 Servicio Inmunología Hospital Infanta Cristina, Badajoz, Spain; 4 Unidad de Patología Infecciosa e Inmunodeficiencias de Pediatría. Hospital Universitari Vall d’Hebron, Universitat Autònoma de Barcelona, Barcelona, Spain; 5 Servicio Inmunología Hospital San Pedro de Alcántara, Cáceres, Spain; 6 Centro de Investigación Biomédica en Red de Enfermedades Raras (CIBERER), Madrid, Spain

**Keywords:** Complement deficiency, Recurrent infections, C3 consumption, Complement factor I, Large deletions, Diagnostic flowchart

## Abstract

**Background:**

Complement Factor I (CFI) is a serine protease with an important role in complement alternative pathway regulation. Complete factor I deficiency is strongly associated with severe infections. Approximately 30 families with this deficiency have been described worldwide.

**Patients and methods:**

We have studied five new Spanish families suffering from CFI deficiency. From 19 screened people, 7 homozygous, 10 heterozygous and 2 healthy subjects were identified. Clinical, biochemical and genetic descriptions are included.

**Results:**

Molecular studies demonstrated 4 novel mutations in the screened individuals; amongst them, we describe here the first great gene deletion reported in the CFI locus, which includes full exon 2 and part of the large intron 1.

**Conclusion:**

CFI deficiency is possibly an underestimated defect and the eventual existence of this deficiency should be tested in those patients exhibiting low C3 and recurrent bacterial infections. We propose a simple diagnostic flowchart to help clinicians in the identification and correct diagnosis of such patients.

## Background

The human complement system consists of more than 35 plasma or membrane-bound proteins whose activation is tightly controlled through the action of complement inhibitors. It is involved in host defense against infectious agents, in the removal of apoptotic cells and immune complexes, and in the modulation of the adaptive immune system [1]. Complement deficiencies, acquired or hereditary, affecting all the soluble components and many of the membrane-anchored receptors and regulators have been described in humans. Most of these deficiencies are inherited as autosomal recessive disorders, with C1 inhibitor (C1INH) and Properdin deficiencies being remarkable exceptions, inherited in autosomal dominant and X-linked manners, respectively. Acquired complement deficiency may be due to infectious diseases or immune complexes-related disorders. Genetic complement deficiencies have been described affecting either protein function or concentration [2,3].

One of the most important complement activation regulators is Complement Factor I (CFI), a serine protease that circulates in a zymogen-like state [4] at a concentration of ~35 μg/mL [5]. The CFI protein is a heavily N-glycosylated heterodimer consisting of two polypeptide chains linked by a single disulfide bond [6,7]. The heavy chain (50KDa) comprises an N-terminal region; an FI membrane attack complex (FIMAC) domain; a CD5 like-domain or scavenger receptor cysteine-rich (SRCR) domain; two low-density lipoprotein receptor (LDLr) domains; and a C-terminal region of unknown function that is a site of sequence variability across species [4]. The light chain (38KDa) contains the serine protease (SP) domain with the conserved catalytic residues [8]. The gene of this complement regulator (CFI, OMIM^*^217030) comprises 13 exons localized on chromosome 4q25 [9,10] and its deficiency is inherited in an autosomal recessive manner.

CFI inactivates C3b by cleaving it into iC3b, C3d and C3dg and, in an analogous way, C4b into C4c and C4d. To properly perform its functions, CFI requires the presence of several cofactor proteins such as C4b-Binding Protein (C4BP), Complement Factor H (CFH), Complement Receptor 1 (CR1/CD35) and Membrane Cofactor Protein (MCP/CD46) [2]. Complete CFI deficiency leads to the uncontrolled amplification of C3 cleavage, resulting in a severe secondary C3 deficiency. Consumption of Complement Factor B (CFB) and CFH are also related with CFI deficiency [2]. Consequently, the most frequently associated pathologies to CFI deficiency are severe infections by encapsulated microorganisms, glomerulonephritis and autoimmune diseases [11].

In this article we describe five new Spanish families with complete CFI deficiency, an underestimated complement defect mainly characterized by recurrent, severe infections, low C3 and normal C4 levels in serum. The fact that this persistent biochemical profile is shared by other relatively infrequent complement abnormalities leads us to propose a simple scheme to help clinicians in the diagnosis of patients presenting this complement phenotype.

## Patients and methods

### Patients

Members from five unrelated Spanish families with CFI deficiency were studied. Serum and DNA samples were obtained under standard conditions after written informed consent of the participants.

Patient I.1 was a 37 year old woman with a long-standing history of recurrent rhino-conjunctivitis. At the age of 4, she had several episodes of otitis, sinusitis and bronchitis. At age 16, she had an episode of meningococcal septicaemia with disseminated intravascular coagulation (DIC) and skin complications. Later, she suffered from an acute arthritis episode. Infections severity declined with age. Her mother (I.2), sister (I.4) and brother (I.5) were also studied.

Patient II.1 was an 8 year old boy, the only child of healthy parents (II.2 and II.3). He had a history of recurrent pneumonia, an episode of meningococcemia and other infections such a balanitis or oral thrush.

Patient III.1 was a 6 year old boy. His parents (III.2, III.3) and brother (III.4) were also studied. He had recurrent episodes of bronchitis. At the age of 5 he had a bacteraemia episode and a meningococcal sepsis with meningitis when he was 6. His recurrent episodes of otitis made the implantation of ear tubes necessary.

Patient IV.1 was a 5 year old girl. She had recurrent episodes of pneumonia and one episode of facial cellulitis. At the age of 5 she had a hypocomplementemic vasculitis episode. Her parents and a sister, aged 8 months, were also studied (IV.2, IV.3 and IV.4).

Patient V.1 was a 6 year old girl with a history of meningitis and pneumonia. At the age of 4, she began suffering from recurrent Henoch-Schönlein purpura outbreaks (15 in two years). Her parents (V.2 and V.3) and her half sister (V.4) were included in the study.

### Complement studies

The functional activity of the classical pathway was measured by CH-50 haemolytic assay, according to standard procedures. C3 and C4 were measured by Nephelometry (Dade Behring, Marburg, Germany). Ouchterlony analysis: Initial screening for the qualitative estimation of the remaining complement components was performed by double immunodifussion with polyclonal antibodies against most of the soluble C components (C1q, C1r, C1s, C2, C5-C9, CFB, CFH and CFI). Serum levels of CFH and CFI proteins were measured by standard methods [12]. Anti CFH auto antibodies (CFHA) were quantified as described by Abarretegui-Garrido et al. [13]. CFB levels were determined by an in-house sandwich ELISA of serum samples by coating the plates with a polyclonal anti-Factor B [14]. The haemolytic assay for C3 nephritic factor (C3NeF) was carried out following the procedures described by Rother [15].

### Western blotting

Four microliters of serum from patients and healthy controls were separated by 8% gel electrophoresis under non-reducing conditions, transferred to a PDVF membrane and detected with polyclonal anti-CFI antibodies, as described by Gonzalez-Rubio et al. [12].

### Sequencing of genomic DNA

The 13 exons and their flanking intronic regions in the CFI gene were amplified and sequenced using primers and conditions described by Ponce-Castro et al. [16]. For mutation nomenclature, the Ensemble accessions ENST00000394634 and ENSP00000378130 were used as reference.

### Multiplex ligation-dependent probe amplification (MLPA)

In order to detect eventual deletions or insertions in the CFI locus, copy number variation was measured with the SALSA MLPA kit P296 aHUS (MRC-Holland, Amsterdam, The Netherlands) following manufacturer instructions. The kit contains probes for the amplification of the 13 CFI exons, their flanking regions and several other reference chromosomal locations. Data normalization and analysis were done with the Coffalyser v9.4 MLPA data analysis software (MRC-Holland).

### Long range PCR (XL-PCR)

A genomic fragment spanning approximately 9Kb (intron 1 to intron 3) was amplified using the GeneAmp XL PCR kit (Applied Biosystems, Madrid, Spain). Reaction products were resolved in 0.7% ultrapure agarose gels, purified with the QIAquick Gel Extraction kit (Qiagen) and sequenced. The sequence of the primers used for long range amplification and sequencing were as follows:

5’GCACCAGTAAGAACCAATCCC3’ (Forward);

5’GAAAGGTTAGGTAATCAAAAAGC3’ (Reverse)

## Results

**Figure 1:**
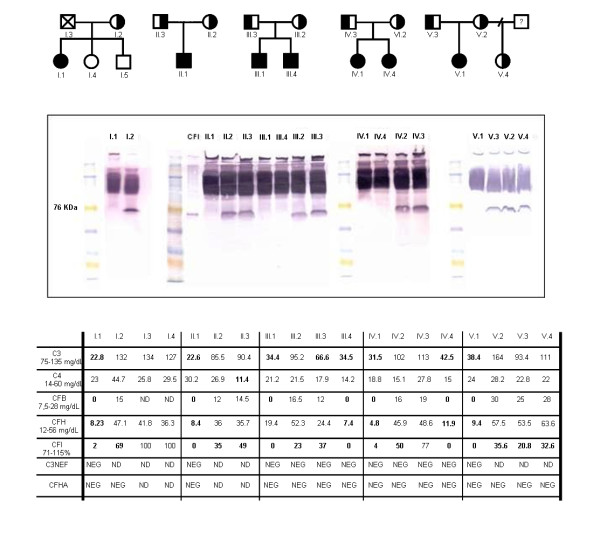
**Pedigrees and complement studies.A**) Pedigrees of the families with CFI deficiency. Black symbols represent complete deficient of CFI and half-filled symbols indicate partial deficiency. **B**) Western blot analysis of CFI in serum samples. **C**) Complement profiles: measurement of C3, C4, CFB, CFH, CFI, C3NeF and CFHA plasma levels. Abbreviations used: NEG, negative. ND, not determined. CFB, Complement Factor B. CFH, Complement Factor H. CFI, Complement Factor I. C3NeF, C3 Nephritic Factor. CFHA, Complement Factor H auto antibodies.

The clinically affected index cases from the five families studied have complete deficiency of CFI protein. Two of the relatives, III.4 and IV.4, brother and sister of III.1 and IV.1 respectively, were also completely CFI deficient, although they had no clinical signs related with this defect by the time the study was performed. All the CFI homozygous deficient had very reduced or no detectable CFI antigen in serum by ELISA and Western Blot (type I CFI deficiency, in which protein production or secretion is below the normal range). Their C3, CFB and CFH levels were also diminished as a consequence of the spontaneous activation and uncontrolled amplification of the complement alternative pathway. In contrast, partial CFI deficiency in some patients’ relatives was characterized by diminished levels of CFI but normal amounts of all other complement proteins analyzed. In all cases, other possible causes of uncontrolled complement consumption, like the presence of C3NeF or anti CFH auto antibodies (CFHA), were ruled out. Complement profiles are shown in Figure 1.

A genetic cause of the deficiency was found in all families. The mutations identified in this series (summarized in Table 1) behave as recessive traits, as only homozygous carriers develop clinical symptoms.

**Table 1:** Summary of the CFI mutations identified in the five families

**Patients**	**Genetic status**	**cDNA** ATG +1	**Protein with signal peptide**	**Exon**	**Domain**	**References**
III.1, III.2, III.4	He	c.266-?_536 + ?del	-	2	-	**Not described**
V.1, V.2, V.4	He	c.80_81delAT	p.D27Afs*18	2	N-terminal	**Not described**
I.1	He	c.485 G > A	p.G162D	4	SRCR	Le Quintrec M. et al. 2008 [17]
IV.1, IV.3, IV4, V.1, V.3	He	c.559 C > T	p.R187X	4	SRCR	**Not described**
II.1, II.2, II.3	Ho He	c.772 G > A	p.A258T	5	LDLRA2	Vyse et al. 1996 [18]
I.1, I.2	He	c.1176_1177dupAT	p.W393Yfs*5	11	SP	Baracho et al. 2003 [19]
III.1, III.3, III.4	He	c.1420 C > T	p.R474X	11	SP	Fremaux-Bacchi et al. 2004 [20]
IV.1, IV.2, IV.4	He	c.1610_1611insAT	p.V537Vfs*2	13	SP	**Not described**

Ho, Homozygote; He, Heterozygote; SRCR, Scavenger Receptor Cysteine Rich domain; LDLRA2, Class A low-density lipoprotein receptor domain 2; SP, Serine. Protease domain.

**Figure 2:**
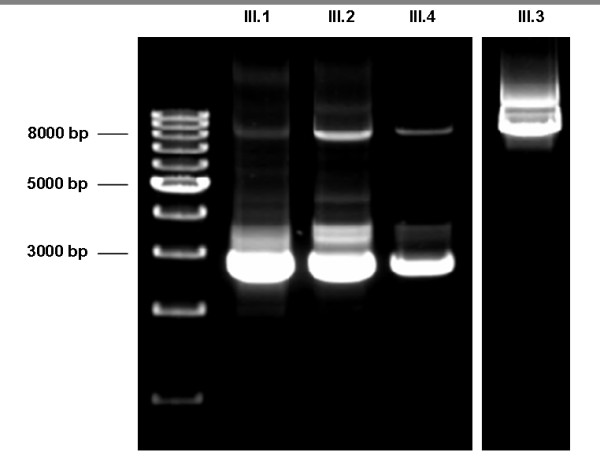
**Long range PCR.** XL-PCR of a genomic fragment spanning approximately 8Kb. Patient III.1, his mother (III.2) and brother (III.4) are heterozygous for the exon 2 deletion. His father (III.3) does not carry the deletion.

We found 4 novel mutations causing CFI deficiency. Among them, we describe here the first large gene deletion reported in the *CFI* locus, which includes exon 2 and part of the very large intron 1. This deletion was found in Family III and was characterized by MLPA. The findings were confirmed by long range PCR (XL-PCR), demonstrating a deletion size of approximately 5-6Kb (c.266-?_536 + ?del) in patient III.1, his mother III.2 and his brother III.4. The resulting fragments were resolved in 0.7% ultrapure agarose gel (Figure 2) but the precise break points of this deletion could not be confidently determined. In addition to that, patient III.2 carries a second, unreported molecular change in exon 4, c.738 A > T; p.N177I. Taking into account that patient III.2’s serum CFI levels are reduced (23% of normal, see Figure 1) but significantly higher than in the homozygous deficient individuals and in her completely deficient offspring (in which CFI is undetectable), the substitution c.738 A > T is probably an unreported polymorphic variant. Patient III.2’s two children inherited the exon 2 deletion but not the c.738 A > T substitution, which implies that both changes are located in different alleles. In any case and due to the lack of co-segregation data, additional studies should be performed to confirm or disprove the polymorphic nature of the c.738 A > T change.

A novel mutation in exon 4 (c.559 C > T) was identified in families IV and V. The C > T transition converted residue R187 into a premature termination codon. No consanguinity was reported affecting families IV and V, but their close geographic origin hints at a founder effect as a probable cause for the co-occurrence of this mutation in these families.

In family IV, a novel AT insertion in exon 13 between nucleotides 1610 and 1611 was also identified. The resultant frameshift change leads to a premature termination codon shortly downstream of the insertion site.

The second mutation found in family V was another unreported mutation. Patient V.I, her mother and half sister, were found to bear an AT deletion (c.80_81delAT) in a region of exon 2 in the N-terminal domain. This deletion converts D27 into an alanine residue and leads to a frame-shifting situation that causes a premature stop codon (p.D27Afs*18).

The remaining mutations found in the patients that we studied had been previously described elsewhere: c.485 G > A [17], c.772 G > A [18], c.1176_1177dupAT [19], c.1420 C > T [20] (Table 1). The second mutation found in patient I.1 (c.485 G > A) is probably a *de novo* alteration, as both brother and sister of the proband do not carry this change. The father was deceased, so no further investigations could be performed on the segregation of this c.485 G > A change.

**Figure 3:**
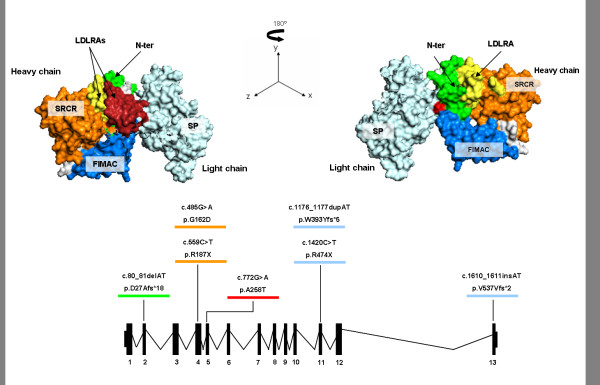
**Schematic model of the CFI protein and gene.** The protein domains, exon/intron gene structure and the mutations found in our series are depicted.

All the mutations reported here are shown in a schematic CFI protein model based on Protein Data Base accession 2XRC (Figure 3).

## Discussion

As part of innate immunity, the complement system plays an important role in the defense against pathogens, especially in early childhood. Spontaneous and continuous activation of the complement cascade [21] must be tightly regulated by both fluid-phase and membrane-bound inhibitors to prevent uncontrolled amplification of C3 cleavage that may result in a state of acquired severe C3 deficiency.

The C3 fragments generated by complement activation are involved in many important functions, such as opsonisation (that enhances microbe uptake into phagocytic cells *via* complement receptors), enhancement of antigen solubility and immunogenicity, non-inflammatory removal of cell debris and immune complexes by phagocytes and promotion of the inflammatory environment required for correct immune response [2,5,22]. Deficiency of Complement Factor I (CFI, OMIM^*^217030) is a very rare primary immunodeficiency disease [23], inherited as an autosomal recessive trait. Heterozygous CFI mutations are associated with atypical haemolytic uremic syndrome (aHUS) [20], a severe disorder characterized by thrombocytopenia, microangiopathic haemolytic anaemia and acute renal failure.

Defective opsonization in these patients makes them susceptible to recurrent pyogenic infections (*Streptococcus pneumoniaeHaemophilus influenzae, Neisseria meningitidis*) as well as to aseptic meningitis [12,24]. Furthermore, deregulation of the complement cascade may result in autoimmune disease [22], vasculitis or glomerulonephritis [25].

Among the CFI deficient individuals reported here, five out of seven homozygotes developed clinical symptoms characteristic of the disease [16,18,26]. Concerning heterozygous carriers, other authors have described symptoms ranging from infections to aHUS [26,27]. To date, none of the relatives with partial CFI deficiency in our series have experienced symptoms.

At the present time, there is no specific therapy for these patients except keeping tight control on their vaccination against common bacterial pathogens such as *S. pneumoniaeH. influenzae* and *N. meningitidis,* and testing their correct antibody production. Despite normal polysaccharide antibody production in these patients, antibody weaning is faster than in the common population [28]. Furthermore, special care has to be taken to prevent associated diseases (using prophylactic antibiotics if necessary) [3]. The main therapeutic goal is to promote the synthesis of anti-capsular antibodies capable of efficiently activating the classical pathway, although plasma infusion may be used in cases of severe infection to ameliorate clinical manifestations.

Familial studies lead us to identify two homozygous children in families III and IV without clinical evidences of CFI deficiency. Their very early diagnoses and the therapeutic precautions taken can most probably explain the lack of clinical manifestations. Even so, we cannot rule out the eventual onset of disease in such patients.

In this study, we aimed to show that in the presence of suggestive pathology and marked, but not absolute, permanent C3 deficiency, screening of complement regulators should be performed. Besides, determination of CFI levels may discard other relatively frequent causes of C3 consumption (like C3NeF or CFH auto antibodies) and thus can guide the clinician in diagnostic tasks. The presence of C3NeF can cause excessive C3 consumption, but clinical signs associated with this autoantibody fit in better with Dense Deposit Disease (DDD) and partial lipodystrophy (PLD) [29,30]. Auto antibodies directed against CFH can also decrease circulating C3 levels while maintaining C4 concentration in its normal range [31-33]. Moreover, functional studies have revealed that autoantibodies provoke the same dysfunction in CFH as mutations in its C-terminal domain, and there seems to be a direct correlation between the extent of CFH dysfunction and the amount of CFH-autoantibody complexes [34]. Recently, anti-CFI autoantibodies have been described in aHUS, but their relationship with the development of the disease remains uncertain [35]. We have not tested the presence of anti-CFI antibodies in these 5 families, although this possibility could be explored in cases in which CFI deficiency is suspected.

**Figure 4:**
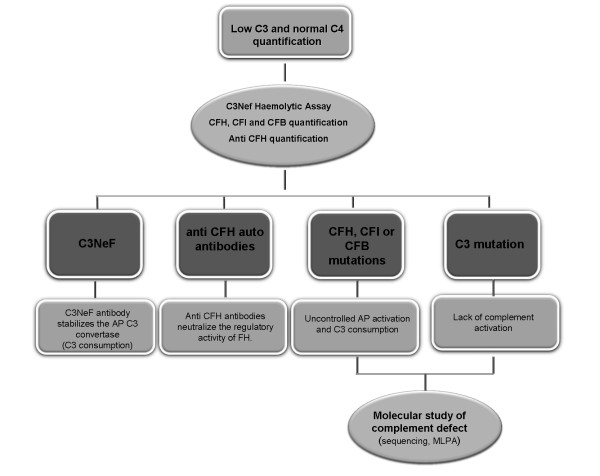
Additional studies for a patient with low C3 (20-30% of normal expression) and normal C4: Alternative pathway of complement activation.

Complete C3 or CFH deficiency presents clinical outcomes similar to those of CFI deficiency so that biochemical and/or functional studies are needed to distinguish among these deficiencies. Identification of the underlying molecular defect can be accomplished following the diagnostic algorithm depicted in Figure 4.

On the other hand, partial defect of C3, CFH or CFI is a susceptibility factor to develop aHUS, one of the most severe human diseases related to alternative pathway deregulation. When CFI deficiency has been established, family studies are needed in order to predict the consequences arising from the segregation of the mutated allele in the family. Multiple concurrent factors, both environmental and genetic, may be necessary in an individual for disease manifestation of aHUS [36]. Environmental factors are thought to initiate endothelium damage, while genetic factors favour disease progression [32]. Moreover, combined deficiencies of two or more complement factors (structural o regulator) have also been described, which adds complexity to these complement deficiencies. Furthermore, a genetic component may also contribute to some typical, infectious HUS cases [33].

## Conclusions

Complement Factor I (CFI) is the major complement inhibitor that degrades activated complement components C3b and C4b in the presence of specific cofactors. Its complete deficiency results in secondary complement deficiency due to uncontrolled spontaneous alternative pathway activation and causes susceptibility to infections. To date, mutations in several CFI domains have been described worldwide. Here, we report four novel mutations and the first large gene deletion in the *CFI* locus.

We propose a general screening for this complement immunodeficiency whenever suggestive clinical features are present in the patient. C3 and C4 quantification are available to most laboratories, thus guiding the clinician at an early stage of the study (see Figure 4). Then, if the results are related to an alternative pathway deficiency with low C3 and normal C4, a multistep process may be performed in a more specialized laboratory for the precise characterization of the defect at the molecular level. Such diagnostic process should include complement functional assays, protein quantification and auto antibody analysis.

## Abbreviations

C1INH, C1 inhibitor; CFI, Complement factor I; LDLr, Low-density lipoprotein receptor; FIMAC, FI Membrane attack complex domain; C4BP, C4b-Binding protein; CFH, Complement factor H; CR1 or CD35, Complement receptor 1; MCP or CD46, Membrane cofactor protein; CFB, Complement factor B; DIC, Disseminated intravascular coagulation; CFHA, Anti CFH auto antibodies; C3NeF, C3 nephritic factor; MLPA, Multiplex ligation-dependent probe amplification; XL-PCR, Long range PCR; AHUS, Atypical haemolytic uremic syndrome; DDD, Dense deposit disease; PLD, Partial lipodystrophy.

## Competing interest

'The author(s) declare that they have no competing interests'.

## Authors’ contributions

MA-D carried out the molecular genetic studies, participated in sequence analysis, performed the immunoassays and drafted the manuscript. AL-L carried out the molecular genetic studies, took part in sequence analysis and drafted the manuscript. SG carried out the molecular genetic studies and immunoassays. IGG, JM, PS-P, CC and PN conceived the study and helped to draft the manuscript. ML-T conceived the study, took part in its design and helped to draft the manuscript. All authors read and approved the final manuscript.
